# Neisseria cinerea-Mediated Peritonitis in an End-Stage Renal Disease Patient on Continuous Ambulatory Peritoneal Dialysis

**DOI:** 10.7759/cureus.20661

**Published:** 2021-12-24

**Authors:** Amarinder Garcha, Sasmit Roy, Raul Ayala, Mamtha Balla, Sreedhar Adapa

**Affiliations:** 1 Nephrology, Kaweah Delta Medical Center, Visalia, USA; 2 Nephrology, Centra Health, Lynchburg, USA; 3 Nephrology, Liberty University School of Medicine, Lynchburg, USA; 4 Internal Medicine, Adventist Health System, Hanford, USA; 5 Internal Medicine, University of Toronto, Toledo, USA

**Keywords:** neisseria cinerea, continuous ambulatory peritoneal dialysis, end stage renal disease, peritoneal dialysis, peritonitis

## Abstract

Peritonitis can be a lethal outcome of peritoneal dialysis (PD), often leading to significant morbidity and mortality. It is caused mostly by gram-positive organisms. Neisseria cinerea is a gram-negative nasal and oropharyngeal commensal, rarely reported as an etiology of peritonitis in PD patients. Our patient was a 37-year-old female on continuous ambulatory peritoneal dialysis for the last seven years, who developed peritonitis found to be from Neisseria cinerea. She didn't respond to broad-spectrum antibiotics well and had to be switched to intermittent hemodialysis. We highlight this important microorganism that can lead to significant morbidity and an unfortunate change in dialysis modality.

## Introduction

Peritonitis is a serious and life-threatening complication of peritoneal dialysis (PD) that can lead to significant morbidity and mortality [1-3]. It is caused mostly by gram-positive organisms [4]. Neisseria cinerea is a gram-negative nasal and oropharyngeal commensal that has been rarely reported as a cause of peritonitis in PD patients [5]. We present a case of peritonitis caused by Neisseria cinerea that is only the fourth such case reported in the literature.

## Case presentation

Our patient is a 37-year-old Caucasian female with a history of end-stage renal disease (ESRD) on continuous ambulatory peritoneal dialysis (CAPD) for the past seven years, tuberous sclerosis, seizure disorder, hypothyroidism, and developmental delay. She presented to the hospital with weakness, shortness of breath, and bilateral lower extremity swelling. She had two previous episodes of peritonitis with coagulase-negative Staphylococcus and Staphylococcus aureus in the preceding six months, which were treated with antibiotics with subsequent negative follow-up peritoneal fluid cultures on both occasions. As per history, ultrafiltration with peritoneal dialysis (PD) had decreased gradually over the last few days. Her medications were aripiprazole 15 mg daily, bumetanide 2 mg twice a day, calcitriol 0.5 ug thrice a week, clonazepam 1 mg daily, and levetiracetam 500 mg twice daily. Her usual peritoneal dialysis prescription was 2.5% dextrose exchange every six hours with a two-liter dwell volume per exchange.

Her vital signs were temperature 97 Fahrenheit (F), pulse rate 97 beats per minute, blood pressure 126/82 mmHg, respiratory rate 18 breaths per minute with oxygen saturation of 98% on room air. Upon physical examination, the patient was in no acute distress. Abdominal examination was unremarkable without any tenderness, and the PD catheter exit site was clean and dry. Laboratory data revealed normal white blood count (WBC), low hemoglobin, and normal platelet count. Serum electrolytes were normal while blood urea nitrogen and creatinine were elevated, as expected (Table 1). The peritoneal dialysate (PD) fluid analysis revealed a WBC count of 2630 cells/microL, with 86% predominant neutrophils. Peritoneal fluid Gram stain revealed >100 WBC, and no organisms were seen.

**Table 1:** Pertinent lab values

TEST (serum)	RESULT	REFERENCE RANGE
White Blood Count (WBC)	6.39 x 10x 3 /mcL	4-10 x 10x 3/mcL
Hemoglobin	7.9 grams/deciliter (g/dL)	12-15 g/dL
Platelet Count	342 x 10x 3/mcL	150-450 x 10x 3/mcL
Sodium	135 millimoles/Liter (mmol/L)	135-145 mmol/L
Potassium	3.9 mmol/L	3.5-5.1 mmol/L
Chloride	98 mmol/L	98-106 mmol/L
Carbon Dioxide	19 mmol/L	23-29 mmol/L
Glucose	102 milligrams/deciliter (mg/dL)	70-105 mg/dL
Blood Urea Nitrogen	83 mg/dL	8-24 mg/dL
Creatinine	8.42 mg/dL	0.7-1.3 mg/dL
Calcium	9.6 mg/dL	8.8-10.2 mg/dL
Albumin	3.4 g/dL	3.4-5.4 g/dL
Peritoneal Fluid WBC Count	2630 cells/microL	< 100 cells/micro L

The patient was started on empiric intraperitoneal vancomycin and ceftazidime. Her PD fluid culture grew Neisseria cinerea. Antibiotics were narrowed down to intraperitoneal ceftazidime alone. Cell count decreased to 100 cells/microL after three days. However, the patient did not improve much clinically. She remained weak and edematous with poor ultrafiltration on PD. Antibiotics were broadened to include gram-positive and gram-negative, as well as fungal coverage with intraperitoneal vancomycin, ceftazidime, and oral fluconazole for a total of three weeks. Repeat cell count from the peritoneal fluid remained normal and peritoneal fluid culture was negative. At this time, the peritoneal dialysis catheter was removed due to ineffective dialysis and the patient was transitioned to hemodialysis.

## Discussion

Peritonitis is a life-threatening serious complication of peritoneal dialysis that can lead to significant morbidity, catheter loss, loss of ultrafiltration, permanent membrane damage, transfer to hemodialysis, and death [1-2]. A retrospective study showed that peritonitis is independently associated with a higher risk of infection-related, cardiovascular, and all-cause mortality in patients who have been on peritoneal dialysis for more than two years [3]. In the United States, a large-scale study observational displayed approximately 62% cases of peritonitis are caused by gram-positive organisms (out of these 31% coagulase-negative staph), 20.5% by gram-negative organisms (equally distributed between E. coli, Klebsiella, Pseudomonas), 3.92% by fungi, and 15.9% reported as culture-negative peritonitis [4]. Among fungal organisms, Candida parapsilosis and Candida albicans are considered the most common agents [6].

Neisseria cinerea is a gram-negative, oxidase-positive, catalase-positive diplococcus that is generally considered a nonpathogenic nasal and oropharyngeal commensal [5]. Neisseria cinerea was first described in 1906, but its strains had subsequently been misidentified as Neisseria catarrhalis [5]. This species was first described in the United States in the year 1984 [5]. There have been case reports of tonsillitis, lymphadenitis [7], and proctitis [8] caused by Neisseria cinerea. It has rarely been isolated as a cause of peritonitis. Prior to ours, there have been three reported cases of peritonitis caused by Neisseria cinerea, two of those were in the same patient two years apart [9-11]. In two of these three cases, the patient did not respond to standard empirical treatment with vancomycin and gentamicin and was effectively treated with ciprofloxacin (intraperitoneal and oral, respectively) [9,11]. A few cases of peritonitis caused by other Neisseria species, such as Neisseria meningitides [12-13], Neisseria sicca [14-16], and Neisseria mucosa [17-18] have also been reported. It is postulated that fastidious organisms, such as Neisseria species, maybe the actual cause of some of the cases of culture-negative peritonitis [11]. Some of these cases of culture-negative peritonitis respond to a combination of broad-spectrum antibiotics such as vancomycin and gentamicin. In such cases, the lack of response to standard first-line therapy should alert clinicians to the possibility of these rare organisms causing peritonitis [11].

In our case, the patient was treated for peritonitis with a standard antibiotic regimen but had to switch the dialysis modality to hemodialysis due to membrane failure.

## Conclusions

We report this case of peritonitis caused by Neisseria cinerea, which is a rare cause of peritonitis in an immunocompetent adult. To our knowledge, this is only the fourth reported case of peritonitis caused by this organism. Through this case vignette, we would like to bring the clinicians’ attention to this organism as a rare cause of peritonitis, as well as treatment options for this scenario.
